# Tuning of the Amount of Se in Rice (*Oryza sativa*) Grain by Varying the Nature of the Irrigation Method: Development of an ICP-MS Analytical Protocol, Validation and Application to 26 Different Rice Genotypes

**DOI:** 10.3390/molecules25081861

**Published:** 2020-04-17

**Authors:** Antonino Spanu, Ilaria Langasco, Massimiliano Valente, Mario Antonello Deroma, Nadia Spano, Francesco Barracu, Maria Itria Pilo, Gavino Sanna

**Affiliations:** 1 Dipartimento di Agraria, Università degli Studi di Sassari, Via E. De Nicola, 1, 07100 Sassari, Italy; ant.spanu@gmail.com (A.S.); mderoma@uniss.it (M.A.D.); fbarracu@uniss.it (F.B.); 2 Dipartimento di Chimica e Farmacia, Università degli Studi di Sassari, Via Vienna, 2, 07100 Sassari, Italy; ilangasco@uniss.it (I.L.); massimiliano.valente@hotmail.com (M.V.); nspano@uniss.it (N.S.); mpilo@uniss.it (M.I.P.)

**Keywords:** selenium, rice, irrigation methods, bioaccumulation, ICP-MS, validation, continuous flooding irrigation, sprinkler irrigation, saturation irrigation, rice genotypes

## Abstract

The amount of specific trace elements like selenium (Se) may be of health concern for humans if contained in too high (or low) quantities in staple foods like rice. Among the attempts aimed to optimize the Se concentration in rice, only few studies have been focused on the use of irrigation methods other than continuous flooding. Since intermittent irriguous methods, like sprinkler and saturation, have found to be effective in modifying the bioaccumulation of arsenic and cadmium in rice kernels, the main goal of this study is to measure the amount of the total Se contained in grains of 26 rice genotypes cultivated for two consecutive agrarian vintages in the same open field and with the same water, but differently irrigated with continuous flooding, sprinkler or saturation. To do this, an original and validated ICP-MS method has been developed. The validation parameters accounted for a high sensitivity and accuracy. Sprinkler irrigation is able to reduce in the average of 90% the amount of total Se in kernels in comparison to values measured in rice irrigated with continuous flooding. In conclusion, different irrigation techniques and rice genotypes seem to be valuable tools in order to allow in the future the customized modulation of the Se concentration in rice grain according to the needs of the various populations.

## 1. Introduction

Selenium (Se) is a trace element in the environment: it ranks 69th on the list of elemental abundances, with an average concentration in the Earth’s crust between 50 and 90 mg kg^−1^ [1]. Although it may be often of concern in terms of terrestrial food-chain contamination, Se is an essential element for humans [2]: it is present in antioxidant enzymes like glutathione oxidases [3] as well as in three of the four thyroid hormones. Furthermore, Se is active in the inhibition of the Hashimoto’s disease [4] as well as in reducing the toxicity of Hg [5]. Insufficient intake of Se can cause Kashin-Beck and Keshan diseases [6], cardiovascular failures [7] and pathologies of the immune and endocrine systems [8]. Nevertheless, intake levels of Se only slightly higher than those showing a physiological action may cause a number of adverse health effects like selenosis [6], whereas the acute intoxication by Se causes gastrointestinal, neurological and respiratory complaints accompanied, in the most serious cases, by cardiac and kidney failures and, only seldom, by death [9]. Although humans may assume selenium both from abiotic and biotic sources, diet is the most important source of intake, and the nature and the amount of the foods assumed modulates the Se assumption. Firstly the National Academy of Sciences of the United States recommended Se intakes between 50 and 200 μg day^−1^ [10] and, later, the World Health Organization set the daily Se dose for an adult person at 30–40 μg day^−1^ [11], while amounts higher than 400 μg day^−1^ (i.e., the Tolerable Upper Intake level for humans) can lead to a number of toxic effects [2]. Due to the fact that a large number of people experiences a monotonous vegetarian diet from foods cultivated in Se-deficient soil areas, the probability of insufficient Se intake for humans largely exceeds that of its overexposure [12], so more than one seventh of the people worldwide is Se-deficient. Anyway, the interval among undesirably high and low intakes of Se is very narrow and, since either overexposure or deficiency in the intake of Se may negatively impact on human health, it is of the utmost importance that the assumption level of this element may be carefully monitored. The amount of Se in soils is reflected in its amount in the foods deriving from them [13]. Se concentration in soils spans over at least two orders of magnitude [3] (i.e., from concentrations even largely less than 0.05 mg kg^−1^ in soils from Finland, New Zealand and China, to amounts higher than 5 mg kg^−1^ in soils from France, Germany, Canada and United States). In addition, the soluble forms of Se (e.g., selenate) are leached from the soil towards both surface and underground water bodies [14]. Furthermore, irrigated agriculture may redistribute and, sometimes, concentrate Se in soils, mainly when water is confined in closed impoundments (e.g., in paddy fields) where the further evaporative concentration might take place [15]. Although Se has never been shown to be essential for higher plants, it is readily assimilated due to its chemical similarity to sulfur [15,16], entering thus in the food chain. The propensity of vegetables for Se bioaccumulation towards edible parts is a function of both soil and climatic factors, plant genotype and nature of the agronomic management. The highest concentrations of Se in foods are expected in protein-rich ones, e.g., fish, meat and eggs for foods of animal origin, and cereals and legumes for those of vegetal origin [16,17]. As a general rule, foods of animal origin (concentration range typically between 0.1 and 1.5 mg kg^−1^) are richer in Se than those of vegetal origin, which concentration ranges between a few tens of μg kg^−1^ and 0.8 mg kg^−1^ [3]. Nevertheless, the highest level of Se in foods (up to 158 mg kg^−1^) was found in a vegetal foodstuff, the Brazilian nut [18].

Rice is the staple food for about the half of the world’s population [19], providing them between 35% and 60% of the dietary calories [20]. It is also the most important cereal with regard to human nutrition [21]. Nevertheless, in recent decades, serious health problems have been ascribed to rice due to its ascertained capability of bioaccumulation of toxic elements like As [22,23,24], Cd [25,26,27] and Hg [28,29,30]. For these reasons, a careful control of the amount not only of such elements, but also of potentially toxic oligoelements (like Se), would be highly advisable in rice. Literature data substantiates that the total Se concentration in rice grain cultivated on unpolluted soils spans over about three orders of magnitude (i.e., between less 2 μg kg^−1^ measured in a Chinese rice [31] and the 2010 μg kg^−1^ found in Nigerian samples [32]). Both the extremes of this very wide range might be harmful for people consuming rice as their staple food. In order to overcome these opposite threats, different methodological approaches have been adopted in order to enhance the Se amount in rice from low-Se soils [33,34,35,36,37], whereas the reduction of the bioaccumulation of Se in rice grain cultivated in Se-rich soils has been accomplished by cultivating low Se-bioaccumulating rice genotypes [38] or by means of phytoremediation techniques [39]. Although the adoption of water management techniques different from the continuous flooding (i.e., the traditional technique of irrigation for rice, still used worldwide) has been proven to be effective in modulating the bioaccumulation phenomena of toxic elements like As [40,41,42,43] and Cd [44,45,46,47] in rice grain, at the best of our knowledge a very scarce attention was until now paid to study the influence of changes in the method of irrigation of rice on its Se concentration [48,49]. Only two pot studies performed first by Li and coworkers [48] and—more recently—by Zhou and coworkers [49] drew attention to the effectiveness of intermittent methods of irrigation (i.e., the so-called “aerobic irrigation” [48,49] and the “alternate flood and aerobic irrigation” [49]) on the bioaccumulation of Se in rice grains. However, the paucity of agronomic details provided in describing both these irrigation techniques, as well as a number of meaningful differences in the relevant experimental plans make the results obtained somehow incoherent among them, if not even contradictory.

The determination of Se in rice has been the object of a number of valuable analytical methods based on different instrumental approaches: among others, it is worthy to cite the voltammetry [50,51], the HR-CS AAS (high resolution continuous source atomic absorption spectroscopy) [52], the ICP-OES (inductively coupled plasma—optical emission spectroscopy) [53], and the INAA (instrumental neutron activation analysis) [54]. Also ICP-MS (inductively coupled plasma—mass spectrometry) methods have been often used for this aim [33,55,56,57,58,59,60,61,62,63] but—unfortunately—their overall reliability is not proven because these methods were often not fully validated.

Our research group, constituted by agronomists and analytical chemists, has been active for years in studying the effects of irrigation methods alternative to the traditional one (i.e., the continuous flooding irrigation, CF) on the bioaccumulation in rice grain of toxic elements like As and Cd. As far as the bioaccumulation of As in rice is concerned, we demonstrated that the adoption of a specific intermittent irrigation (i.e., the sprinkler irrigation, SP) is able to reduce by 98% (average value on 37 different rice genotypes) the concentration of As in rice grain in comparison to values measured in the same samples irrigated by CF [43]. At the best of our knowledge, this is the highest lowering of As concentration in rice grain, obtained with any technique, until now reported in literature. More recently, we reported the dramatically opposite behavior that two intermittent methods of irrigation apparently similar among them (i.e., the aforementioned SP irrigation and the saturation irrigation, SA) can exercise on the Cd bioaccumulation on rice grain. In a field experiment (26 different rice genotypes cultivated along two consecutive agricultural years), the average Cd amount in rice grain irrigated by SP is 20% less than that the corresponding amount measured in rice irrigated by CF, whereas rice irrigated by SA substantiates an outstanding average increase of 900% of the Cd amount vs. rice irrigated by CF [47]. Hence, following our interest for the study of the influence of alternative methods of irrigation for rice on the bioaccumulation phenomena of elements of potential health concern for humans, we think deserving of interest to ascertain if the adoption of intermittent methods of irrigation may play a role in modulating the amount of Se in rice grain. To reliably do this, we planned to develop, to validate and to apply to several tens of real samples a specific ICP-MS method devoted to measure the total Se amount in rice grain.

## 2. Results

### 2.1. Methods Assessment

Due to its very high sensitivity and its straightforward linear working range, that spans up to nine orders of magnitude (from the pg dm^−3^ to the mg dm^−3^ levels), the ICP-MS technique has a significant potential to be employed for many trace element applications, and for this reason it has been previously chosen by a number of different research groups with the aim to measure the concentration of oligoelements like Se in rice grain. As a matter of fact, however, the adoption of one of the literature methods for this study has been prevented by the substantial absence of a sufficient set of validation parameters. On these bases, the need of a fully validated ICP-MS method aimed to measure in a reliable way the amount of the total Se in rice grain has been fulfilled assessing and validating a sensitive and accurate original method. In this framework, also a new ICP-MS method aimed to measure the extractable amount of Se from soils (by means a HNO_3_/HCl disgregation) has been developed and validated.

One of the most significant choices in order to assess a new ICP-MS method for the determination of traces of Se in rice grain is the analytical mass. Se has six natural isotopes (^74^Se, ^76^Se, ^77^Se, ^78^Se, ^80^Se and ^82^Se); among them, those showing the highest abundance—and for this in principle most suitable for trace analysis—are ^80^Se (49.80%), ^78^Se (23.69%) and ^76^Se (9.37%). Unfortunately, all of them are heavily interfered by Ar_2_^+^ ions (^36^Ar^40^Ar^+^ on ^78^Se,^−38^Ar^40^Ar^+^ on ^78^Se, and ^40^Ar_2_^+^ on ^80^Se) and, due to their huge abundance in the plasma, these isotopes have been not kept into consideration for this method. Hence, a good choice was represented by ^82^Se (relative abundance: 8.73%). In this case, the only Ar-based possible interference was that of the triatomic molecular ion ^40^Ar_2_^1^H^+^, but the possibility to form this in the plasma is significantly lower than those of the only-Ar molecular ions. Beyond the possible—but not significant, due to their very low abundance in this matrix—interference of double charge lanthanide ions (i.e., Ho^2+^, Gd^2+^ and Er^2+^), the only reliable spectral polyatomic interference was that of ^81^Br^1^H^+^ and that of the isobaric interference by ^82^Kr (relative abundance: 11.59%). However, the NexION 300X ICP-MS is provided by a collision cell aimed to reduce the extent of the polyatomic interferences, mainly those by molecular ions with a large ionic radius, like Br. In this framework, He is a reliable choice to perform a kinetic energy discrimination (KED) in a collision cell. This gas, light in mass and inert, can flow in the collision cell slightly increasing the pressure in it. Bombardment with He atoms of large molecular ions will cause them to lose significantly its energy (much more that happens to the smaller single ions of analyte) and then a bias voltage at the cell exit can easily exclude them from the ion beam. In this study the optimized He flow on the KED cell was set to 3.50 dm^3^ min^−1^; i.e., a compromise between the highest suppression of both the interferences of ^81^Br^1^H^+^ and ^40^Ar_2_^1^H^+^ molecular ions and the minimization of loss of the ^82^Se^+^ analyte ion. On the other hand, the adoption of a specific equation of correction has been able to keep into account (and hence subtract it from the *m*/*z* 82 gross signal) the contribution of the ^82^Kr^+^ ion on the analyte signal.

### 2.2. Validation

Validation of the developed method has been accomplished in terms of LoD, LoQ, linearity, precision and trueness. Table 1 reports the features describing the performances of the method here considered.

The proposed method is characterized by a very low LoD (0.73 μg kg^−1^), measured according Currie [64], and an excellent linearity within the experimental interval of concentration (i.e., two orders of magnitude starting from the LoQ), although the linearity can be easily extended up to the mg kg^−1^ level without any significant worsening of the determination coefficient R^2^. In addition, the casual distribution of the residuals of the regression line allowed to exclude the presence of any “hidden” deviation from linearity. The precision and the trueness were always measured on different CRMs of rice flour, where the certificated Se amount ranged from 61 μg kg^−1^ and 380 μg kg^−1^. Precision was evaluated both in terms of repeatability and intermediate precision. As a function of the nature of each CRM, the CV of repeatability ranged between 2.4% (NIST 1568a) and 12% (NCSZC 11007), whereas the CV of intermediate precision ranged between 7.0% (NCSZC 73008) and 20% (IRMM 804). All precision parameters have found to be acceptable according to the Horwitz’s theory [65]. Finally, trueness was evaluated by three repeated analyses of the two CRMs’ rice flour which certificated the concentration of Se, i.e., NIST 1568a (certified Se concentration = 380 ± 40 μg kg^−1^) and NCSZC 73,008 (certified Se concentration = 61 ± 15 μg kg^−1^). A quantitative recovery (criteria: two tails t-test, *p* = 0.95) has been accounted for both CRMs, substantiating hence the absence of any bias for the proposed method.

### 2.3. Se in Rice Grain

The concentrations of Se measured in rice grain along the study are reported in Table 2.

### 2.4. Se in Soils and Irrigation Waters

The average concentration of Se in the soils (layer depth: 0–20 cm, particle size: < 0.2 mm) measured along the two years of experimentation used in the study as a function of the irrigation method (*N* = 6) is 0.6 ± 0.2 mg kg^−1^ for CF, 0.6 ± 0.2 mg kg^−1^ for SA and 1.0 ± 0.2 mg kg^−1^ for SP, respectively. The absence of any bias of the method described in the experimental section has been successfully ascertained (*p* = 0.95) by analyzing three different aliquots of the SS-1 EnviroMAT contaminated soil (Se certified amount: 0.780 ± 0.140 mg kg^−1^, Se found: 0.8 ± 0.1 mg kg^−1^). In addition, the average concentration of Se in ten aliquots of irrigation waters sampled monthly in the May-September time spanned along the two years of experimentation is 0.4 ± 0.2 μg dm^−3^.

## 3. Discussion

Literature studies aimed to manage the amount of Se in rice grain were mainly focused towards: (i) its enrichment, by means of the soil supplementation with Se-containing aqueous solutions, or (ii) its reduction, as a consequence of the application of phytoremediation techniques to high-Se paddy fields or by means of a proper choice of low-Se bioaccumulating rice genotypes. The fact that rice was always irrigated worldwide by means of the continuous flooding technique is likely a good rationale for explaining how, until now, the water management techniques have been sparingly kept into consideration as a possible tool aimed to modulate the Se bioaccumulation in rice grain. At the best of our knowledge, only two pot studies dealt with this topic. Firstly, the contribution of Li et al. [48] is relative to one rice genotype irrigated with flooded or aerobic techniques, and subjected to (i) no addition of Se to soil or (ii) soil supplementation with a 0.5 mg kg^−1^ of Se as Na_2_SeO_3_ or (iii) soil supplementation with a 0.5 mg kg^−1^ of Se as Na_2_SeO_4_, respectively. Unfortunately, no description able to replicate in forthcoming experiments the aerobic technique of irrigation has been provided in this contribution. Rice growth in soil not supplemented with Se shown a reduction of the amount of Se in rice grain passing by flooded irrigation to aerobic irrigation, whereas the opposite happens when the soil is supplemented with both aqueous solutions of Se. In particular, the Se bioaccumulation in rice grain irrigated aerobically seems largely higher on soil supplemented with selenate rather than in that supplemented in selenite. More recently, the study of Zhou et al. [49] (one rice genotype, soil supplemented with Na_2_SeO_3_ up a final concentration in Se of 0.5 mg kg^−1^ and three water management techniques, i.e., the traditional flooded irrigation and two intermittent techniques) confirms the tendency to increase of Se bioaccumulation in rice grain when aerobic irrigation was used, while flooded irrigation is effective in minimizing the concentration of Se in kernels. Albeit the undoubted interest in these results, their application in a real situation looks quite questionable, due to some specific features of their relevant experimental plans. Firstly, both studies focused on only one rice genotype, while the extreme variability of Se bioaccumulation at varying of the rice genotype is well known in literature [37,38]. Furthermore, the applicability of these results looks unlikely in a real agronomic context, because of the difficulty to attain in the open field the same level of control of the key variables reachable in a pot experiment. Finally, the details of the intermittent methods of irrigation used in both studies as an alternative to the continuous flooding one are not carefully described in order to allow their reproducibility in other studies.

Hence, this study has been specifically planned in order to overcome these aforementioned issues. Twenty-six different rice genotypes, belonging to two of the most representative subspecies (i.e., Indica and Japonica) have been selected, cultivated for two consecutive years in three unpolluted and not Se-supplemented open fields belonging to the same site, and irrigated with unpolluted water with three different and carefully described water management methods. Data reported in Table 2 substantiate that the Se amount measured in this experimentation spans over a quite wide range of concentration (i.e., between 150 μg kg^−1^ (Carnise, CF, Year 1 and Gloria, SA, Year 2) and 5 μg kg^−1^ (CRV 108 and CRV 390, SP, Year 1)), with an average value in the overall experiment of 65 μg kg^−1^.

In particular, while the amounts measured in rice samples irrigated with both CF and SA water management methods are well within the concentration range of total Se measured in rice grain produced [37,38,51,55,57,66] and marketed [56,58,66,67,68,69] worldwide, almost all the amounts measured in kernels irrigated by SP are lower than such range. It is a matter of fact that, whereas the average amount measured along the two years experimentation in all rice samples irrigated by CF and SA methods is 83 μg kg^−1^ and 103 μg kg^−1^, respectively, the two-years average concentration of the same 26 rice genotypes irrigated by SP is only 9 μg kg^−1^. To the best of our knowledge, this is the first time that such an outstanding reduction of the Se bioaccumulation has been achieved by only changing the water management method, and this is more unexpected because a previous study on the same topic [48] led to diametrically opposed conclusions (i.e., both the intermittent methods of irrigation used in it have caused, in comparison to the Se amounts measured in rice kernels irrigated in the traditional way, a rough doubling of the concentration of such element). The differences among the Se amount in rice cultivated in the three water management methods here considered are reflected also in their concentration range measured along the two years of experimentation. Whereas the range measured for rice irrigated by SA is within that measured for rice irrigated by CF (i.e., between 130 and 63 μg kg^−1^ and between 150 and 55 μg kg^−1^, respectively), the range measured is largely below the range of rice irrigated by SP.

The topic of the differences in Se accumulation on rice kernels at varying of the genotype is also seldom treated in detail. At the best of our knowledge, only the contribution of Zhang and coworkers [38] is worthy to cite: the analysis of 151 rice grains genotypes, all belonging to the Japonica subspecies and grown in the same soil, showed a total Se concentration spanning between 29 μg kg^−1^ and 103 μg kg^−1^. Both the range of Se in rice grain measured in our experiment, relative to 26 genotypes (20 belonging to the Japonica subspecies and 6 belonging to the Indica subspecies) growth in the same soil and irrigated with CF and SA, are comparable with those measured by Zhang et al., whereas the range observed in the same genotypes irrigated by SP is not, because it is shifted towards very low concentration of Se.

The relative tendency of each rice genotype irrigated with a stated water management method to bioaccumulate Se in kernels has been measured in terms of Se mean normalized bioaccumulation ratio (MNBR), i.e., the mean value (on both years of the experimentation) of the normalized bioaccumulation ratio of Se, calculated as follows: (C_Se i_ − C_Se m_)/C_Se m_, where C_Se i_ is the Se concentration for the i-th rice genotype, and C_Se m_ is the mean value of the Se concentration for all rice genotypes irrigated with a stated method in a stated year of experimentation. Hence, a negative value of Se MNBR is representative of a genotype, which, in the two years of experimentation, provides an average Se amount in rice grain that is less than that of the relevant mean concentrations. Furthermore, it is interesting to consider also the range between the Se NBR measured in both years of experimentation for a stated genotype and a stated irrigation method (i.e., CF RANGE, SA RANGE and SP RANGE), useful for measuring the dispersion of the data along the experimentation. For this reason, low values of this parameter support, for a certain genotype irrigated with a stated method, a constant behavior in the Se bioaccumulation in rice grain, whereas the opposite is true for genotypes showing high RANGE amounts.

**Figure 1:**
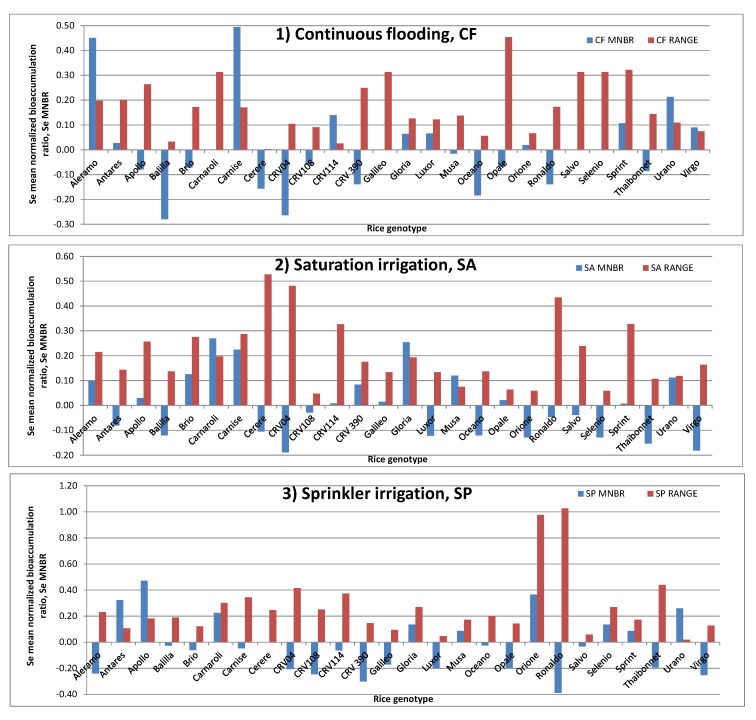
Behavior of the mean normalized bioaccumulation ratio of Se (Se MNBR) and range of the normalized bioaccumulation ratio of Se (RANGE) along two years in rice grains of 26 genotypes irrigated (**1**) by continuous flooding, CF; (**2**) by saturation, SA and (**3**) by sprinkler, SP.

Figure 1 presents the behavior of both the MNBR and of the RANGE parameters for all rice genotypes. As a general behavior, the highest values of Se MNBR have been found when the CF method has been used, whereas the lowest values of such parameter corresponded to the SP method. Giving a closer look to the data reported in Figure 1, the 26 rice genotypes can be divided into three major groups according their MNBR values: (i) those that were constantly bioaccumulators of Se in grain; (ii) those not bioaccumulating Se in the grain and (iii) the remaining genotypes, which show a mixed behavior as a function of the irrigation method used. To the first group belong genotypes like Gloria, Sprint and Urano, whereas the second, more numerous than the first, is represented by Balilla, CRV04, CRV108, Oceano, Ronaldo and Thaibonnet. The genotypes belonging to the first group are actually constant, but slight bioaccumulators of Se. Whereas the amount in rice grain does not vary significantly as a function of the agrarian vintage for Gloria and Urano rice genotypes, the same does not happen for the Sprint genotype, mainly when the CF and the SA irrigation techniques were used. Concerning also the six rice genotypes bearing always scarce capabilities of Se bioaccumulation in kernels, the Balilla and Ronaldo ones show the greatest tendency to minimize the bioaccumulation of Se when using the CF and the SP irrigation methods, respectively, whereas the remaining four genotypes show Se MNBR values always below −0.3 units.

On the other hand, the very high values of RANGE noted for the Ronaldo and CRV04 genotypes in the SA and SP irrigation methods, and for Thaibonnet in SP irrigation account for a wide variation of Se bioaccumulation from year to year. Among the third group of genotypes, it is possible to substantiate the great capability of Aleramo and, mainly, of Carnise varieties to bioconcentrate Se when CF irrigation was used, whereas Antares, Apollo and Carnaroli varieties are moderate bioconcentrators of Se in SP irrigation. The Carnaroli genotype is the highest bioconcentrator of Se when the SA irrigation method was used. The remaining genotypes show a behavior not far from the average values of Se MNBR and RANGE, with the sole exceptions of Opale, Cerere and Orione varieties, which show a large variability of the RANGE if irrigated by means CF, SA and SP methods, respectively.

The effect of the subspecies on the Se bioaccumulation is not well defined. Among the six genotypes belonging to the Indica subspecies, two (i.e., Sprint and Urano) are always high bioaccumulators of Se, other two (i.e., Oceano and Thaibonnet) are always low bioaccumulators of Se, whereas the last two (i.e., Apollo and Salvo) show a mixed behavior. On the whole, it is possible conclude that the probability that a Japonica genotype might have an intermediate behavior with respect the Se bioconcentration as a function of the irrigation method is reasonability higher than that for a genotype belonging of the Indica subspecies.

Finally, no statistically meaningful difference in the Se bioaccumulation in rice grain is observed by varying the agrarian vintage. It is possible to observe a general reduction of the Se amounts in rice irrigated by CF passing from the first year to the second year of experimentation, and a corresponding increase of Se concentration in rice kernels obtained by irrigations accomplished with intermittent methods (i.e., SA and SP). Nevertheless, the very high values of the standard deviation observed prevented us to formulate any reliable hypothesis among a possible effect ascribable of the agrarian vintage in the Se bioaccumulation in rice grain. In this context, it should be emphasized that the high values of the standard deviation is almost completely due to the very wide difference in the Se amounts measured in the four different physical samples that concur to each analytical data reported in Table 2, and not to an inaccuracy of the analytical method, as clearly shown from precision data reported in Table 1.

## 4. Materials and Methods

### 4.1. Site, Soils, Irrigation Water and Rice Genotypes

Rice cultivation was held at the University of Sassari’s experimental farm “Santa Lucia”, near Zeddiani (OR), Sardinia, Italy (39°59′ N, 8°40′ E; 15 m AMSL). In the site chosen for the experiment the climate is Mediterranean, characterized by rare precipitations (always less than 150 mm in the period May-September) and quite high average temperatures in the rice-growing season (between 15 °C—i.e., the average minimum temperature in May—and 30 °C—i.e., the average maximum temperature in July and in August). The soil is a *Typic Eutric Haplic Fluvisol*, according to the World Reference Base for Soil Resources. The areas used for our study’s cultivation with CF and SA irrigation have been continuously used in this way for last 35 years. The soils used for cultivating rice with SP irrigation are clayey with good water retention capacity. The fields were chosen based on the similarity of both their hydrological and chemical properties as well as their pedological classification. Table 3 reports the chemical and hydrological characterization of the soils chosen for the experiment. In summary, they have—in their surface horizons—a sandy-clay texture, a subalkaline pH, a negligible amount of carbonates, a low amount of organic carbon and total nitrogen, whereas the amounts of assimilable phosphorus and exchangeable potassium were sufficient for the requirements of the cultivation of rice.

Water used for all irrigation methods was taken from Lake Omodeo, i.e., the largest artificial basin of Sardinia. Reliable samples of such water were collected monthly, acidified with HNO_3_ up to pH = 2 and stored in a polyethylene bottle at the temperature of +4 °C until analysis. Twenty-six genotypes were cultivated in both years of the experiment (20 from the Japonica subspecies and six from the Indica subspecies), and all rice genotypes were grown in three adjoining fields (one for each irrigation method) inside the experimental farm.

### 4.2. Description of Irrigation Methods

The main features of the water management methods used have been reported below, whereas their extensive description has been reported elsewhere [47].

#### 4.2.1. Continuous Flooding Irrigation, CF

CF is the almost ubiquitous irrigation technique used in rice farming around the world. The paddy field (that must be perfectly flat in its whole extent) was leveled and surrounded by embankments to contain irrigation water. Rice plants were kept flooded with a constant blanket of ca. 10 cm of water for almost the entire growing season (i.e., from seeding to 10–15 days before harvest). The flooding conditions were held until the late-ripening cultivar had reached its full ripening. In these conditions there is a constant prevalence of reductive conditions in the soil, as supported by its constantly negative values of the redox potential [43,47].

#### 4.2.2. Sprinkler Irrigation, SP

The sprinkler irrigation method (SP) is one of the intermittent water management systems. It was optimized for rice in Sardinia, Italy, [72,73] where its adoption in place of CF, applied to several tens of rice genotypes over a number of crop years, has produced no significant differences in yields [43]. When SP irrigation was used, the soil was never flooded. Water was sprayed into the air by means of low- (or medium-) flow sprinkler heads and letting it fall on the soil as rainfall. Water quantities provided in each irrigation cycle were determined on the basis of the evapotranspiration amounts (normally form every 2–3 days to every 4–5 days, depending on the phenological phases of rice and by the meteoclimatic conditions [47]). Since SP irrigation keeps the soil close to its field capacity (i.e., when only half of the volume of the pores in the soil is occupied by water, whereas the remaining half of the pores is occupied by air) [74,75], the layer explored by roots is always in oxidized conditions, as demonstrated by the always positive value of the redox potential [43,47]. The adoption of this technique in place of CF allows to reach both for agronomic and environmental advantages: (1) the water requirements are greatly shortened (at least the 50% of water savings); (2) the control of weeds is made easier and no specific agricultural machinery (i.e., a crawler tractor or a crawler harvester) is required, due to the fact that the soil is never flooded; (3) no preliminary (and sometimes very expensive) leveling of soils is required; (4) the rice cultivation is possible also in sloping soils: since no specific orography of the rice field is required using SP, the amounts of soils potentially eligible for rice cultivation should be enhanced, as well as the net production of rice grain, and also the crop rotation regime would be easier (it is quite common that the paddy fields can be used for rice cultivations for many consecutive decades, and this cause in it a continuous depletion of nutrients and a constant accumulation of toxic elements and molecules); (5) it is possible to reach energy (and money) savings because of (i) no leveling of soil is needed; (ii) no specific machinery for rice cultivation is needed; (iii) the number (and the strength) of both the fertilization and herbicide treatments have been reduced. Finally, also the emissions of greenhouse gases (a very high production of methane has been substantiated by the paddy fields in the CF irrigation [76]) should be minimized.

#### 4.2.3. Saturation Irrigation, SA

SA is another intermittent method of irrigation, of which main features are intermediate between CF and SP. As that happens for SP, also in this case the soil was never flooded; it was cyclically saturated whenever the top 5 cm has been dry (usually this happens, in the latitude of the experiment, once each 5 and 7 days, depending on both the phenological phases of the species and the meteoclimatic conditions). The field must be leveled—as with CF—but perimetral embankments are not required, because it is never flooded. The amount of irrigation water provided for each cycle in SA was equal to the soil’s water storage capacity: in this condition, all soil pores are filled with water, but no water pond is visible in the field as a consequence of the irrigation. To allow a homogeneous irrigation in the field, it has surrounded by 10 cm-deep furrows. Water flows in the furrows and saturates the field. By using this irrigation method, evaporation and percolation losses are minimized, and the water saving achieved are between 25 and 30% of the amounts required for CF irrigation. The redox potential of soil irrigated by SA follows a roughly cyclical behavior between two consecutive irrigations. Just after the saturation has been accomplished, the redox potential is very close to values typical of a continuously flooded soil. Starting from this point, the redox potential of the soil continuously rises, up to become, just before the following irrigation, much more positive than the values usually measured in SP (e.g., up to + 370 mV vs. SCE [47]).

### 4.3. Experimental Design

All rice genotypes were cultivated in proximal fields (one for each irrigation method considered). The surface of each field was 3000 m^2^ in both the years of the experiment. The experimental design was a randomized block with four replications for each genotype. The surface of each replication parcel was typically of 10 m^2^. In each parcel, two rows 2 m in length was selected as representative of the parcel, with the aim to measure with it a number of biological and agronomic parameters like the number of plants at emergence stage, the number of shots and the number of fertile panicles.

### 4.4. Crop Management

The assessment of the seed bed was accomplished first by chisel plowing to a depth of 20 cm, and later by a secondary tillage by means of a field cultivator. A preliminary leveling of the fields irrigated by CF and SA was accomplished with a laser-guided leveler, while the field irrigated by SP remained unleveled. Sowing was always performed on dry soil by means a seed drill (sowing depth: 3 cm; inter-row distance: 14 cm; sowing density: 500 viable seeds m^−2^). Adherence between soil and seeds was optimized by taxiing the sown field with a grooved roller; in this way it is also possible break up smaller clumps, improving hence the effectiveness of pre-emergence weed control. Table S1 of the Supplementary Materials reported, for each irrigation method and each year of experiment, fertilization and herbicide treatments used in this study.

### 4.5. Harvesting and Sampling of Rice

Depending on the irrigation method harvesting was accomplished, for all rice genotypes, on the same day (between the last days of September and the first days of October). Yield was measured, for each genotype and each sub-plot, using a small plot combine harvester. Furthermore, for each sub-plot, 100 g of paddy rice was dried at 32 °C, mechanically husked and bleached, and then analyzed by ICP-MS.

### 4.6. Instrumentation

Rice and soil samples were desegregated by acid/oxidant-assisted microwave irradiation using a microwave oven Ethos Easy Lab Station from Milestone, Sorisole, Italy. The Se determination was always performed using a NexION 300X ICP-MS spectrometer (Perkin Elmer, Milan, Italy), equipped with a nebulization system composed of a glass concentric nebulizer, a glass cyclonic spray chamber, an autosampler model S10 and a KED collision cell. The ICP-MS spectrometer was controlled by a proprietary software (NexION software Version 1.0) running under the Windows 7 environment. The Eh potentials of the soils were measured using a Thermo Orion model 210A electronic millivoltmeter, connected to a Pt electrode and an Ag/AgCl reference electrode (Amel Instruments, Milan, Italy).

### 4.7. Reagents

The 67% aqueous solution of HNO_3_, the 37% aqueous solution of HCl and the 30% aqueous solution of H_2_O_2_ were all Normatom reagents from VWR (Milan, Italy), and were used in all the phases of the study. A 2% HNO_3_ standard aqueous solution of Se (1000 mg dm^−3^) was purchased from Fluka, Milan, Italy. High-purity water (type I, resistance > 18 MΩ) was produced using a MilliQplus System (Millipore, Vimodrone, Italy) and was used for all of the analytical phases of the study. The ICP-MS Setup Solution (a 1% (*v*/*v*) aqueous solution of HNO_3_ containing 1 μg L^−1^ each of Be, Ce, Fe, In, Li, Mg, Pb and U), the KED Setup Solution (a 1% (*v*/*v*) aqueous solution of HNO_3_ containing 10 μg L^−1^ of Co and 1 μg L^−1^ of Ce) and the internal standard solution (containing 10 μg dm^−3^ of Rh in a 1% (*v*/*v*) aqueous solution of HNO_3_) were all from Perkin Elmer. Four certified rice flours were used in this study: the NIST SRM 1568a (National Institute of Standards and Technology, Gaithersburg, MA, USA), the IRMM 804 (European Commission Joint Research Centre, Institute for Reference Materials and Measurements, Geel, Belgium), NCSZC 73,008 and NCSZC 11,007 (both produced by the China National Analysis Centre, Beijing, China), whereas the certified soil SS-1 EnviroMAT contaminated soil was produced by SCP Science (Baie D’Urfé, QC, Canada).

### 4.8. Analytical Methods

Literature methods were adopted to measure the Eh potential, the chemical and hydrological parameters of soils [74,75], and the amount of Se in irrigation waters [77], whereas original and validated methods were used to measure the total concentration of Se in rice grain and the extractable amount of Se in soil samples.

#### 4.8.1. Disgregation of Rice Samples

Disgregation of rice samples was accomplished by means a microwave-assisted acid/oxidant mineralization. The microwave oven contains a sectored basket consisting of fifteen perfluoroalkoxy alkane (PFA) vessels. Each disgregation cycle consisted hence of twelve rice samples, two method blanks, and one certified reference material. For this purpose, NIST SRM 1568a (certified Se = 380 ± 40 μg kg^−1^) and NCSZC 73,008 (certified Se = 61 ± 15 μg kg^−1^) certified rice flours have been used. Ca. 1.4 g of the rice grain sample was weighed on an analytical balance (accuracy ± 0.0001 g) in a PFA vessel and then treated with 4 cm^3^ of 67% HNO_3_, 2 cm^3^ of 30% H_2_O_2_ and 4 cm^3^ of H_2_O. After closing, the basket underwent a cycle consisting in (i) the heating in 10 min of the solution inside each vessel up to 180 °C; (ii) the disgregation of the organic substance, allowing the solution to stay at 180 °C for 15 min; (iii) the cooling of the solutions, reaching the temperature of 70 °C in 15 min. After extracting from the oven, the whole basket was then cooled in an ice/acetone mixture until reaching −20 °C before opening. Then, the solution was quantitatively recovered, filtered off in a 0.22 μm pore size polypropylene filter and diluted with water until reaching a final volume of 50 cm^3^.

#### 4.8.2. Disgregation of Soils

Also in this case the disgregation of soils was accomplished by a microwave-assisted acid/oxidant mineralization. Twelve samples, two method blanks, and one certified reference material constituted the typical basket for the mineralization of soils. In this case, a SS-1 EnviroMAT (Se = 780 ± 140 μg kg^−1^) certified soil has been used. Ca. 0.5 g of the soil sample (particle size < 0.2 mm) was weighed on an analytical balance (accuracy ± 0.0001 g) in a PFA vessel and then treated with 9 cm^3^ of 67% HNO_3_ and 3 cm^3^ of 37% HCl. Since the use of HF in the disgregation phase has been deliberately discarded in order to prevent the presence of free HF in the final solution, the Se concentrations measured in this study should be intended as an “extractable” fraction, due to the lacking of the fraction of this element still blocked in the undissolved silicate matrix. After closing, the basket underwent a cycle consisting in (i) the heating in 10 min of the solution inside each vessel up to 180 °C; (ii) the disgregation of the matrix, allowing the solution to stay at 180 °C for 10 min; (iii) the cooling of the solutions, reaching the temperature of 70 °C in 10 min. After extracting from the oven, the whole basket was then cooled in an ice/acetone mixture until reaching −20 °C before opening. Then, the solution was separated from the undissolved solid residue, quantitatively recovered, filtered off in a 0.22 μm pore size polypropylene filter and diluted with water until reaching a final volume of 500 cm^3^.

#### 4.8.3. Pre-Treatment of Irrigation Water

The samples were allowed to reach the room temperature, adjusted with HNO_3_ up a 1% final concentration, filtered off in a 0.22 μm pore size polypropylene filter and immediately analyzed.

#### 4.8.4. ICP-MS Determination of Se in Disgregated Matrices and Irrigation Water

The optimized parameters used during the ICP-MS measurements are: RF generator power output: 1600 W; argon flows: plasma, 17.995 dm^3^ min^−1^; nebulizer; 0.991 dm^3^ min^−1^, auxiliary 1.203 dm^3^ min^−1^; KED gas, helium, at flow 3.500 cm^3^ min^−1^; optimization on masses of ^7^Li, ^89^Y, ^205^Tl; data acquisition: dwell time of 50 μs, 3 points per peak, acquisition time of 3 s; analytical mass, ^82^Se. A correction equation was used to compensate the isobaric interference due to ^82^Kr. Unless otherwise stated, each analytical sample was analyzed three times, and each analytical datum is the average of five replicated ICP-MS measurements. No significant statistical difference (criteria: two-tail t-test, *p* = 0.95) have been found between the slopes of the regression lines obtained using external calibration and multiple standard additions on a real sample in the concentration interval between the limit of quantification and 10 μg L^−1^. For this reason, quantification was always accomplished using the external calibration. A 10 μg dm^−3^ Rh solution as an internal standard in order to compensate any possible signal instability, whereas a washing cycle of at least 30 s was settled between two subsequent samples with the aim to eliminate any memory effects. All reported data were blank corrected. In order to monitor constantly the overall accuracy level of the method, a blank was run up every four samples, a 0.5 μg L^−1^ Se standard solution was run every eight samples, and a CRM mineralized solution (an aliquot of NCSZC 73,008 for rice samples and an aliquot of SS-1 EnviroMAT for soil samples), was run every twelve samples.

### 4.9. Statistical Analysis

A two-tail t-test at (*p* = 95%) was used in ascertaining the existence of matrix effect in the method and in the evaluation of bias in the trueness evaluation.

## 5. Conclusions

Rice is the staple food for roughly half of mankind, and Se is one of the most representative oligoelements in foods. Since also low variations out the amounts of Se physiologically needed for humans can cause serious health issues, a careful control of the concentration of Se in rice grain seems to be an issue of the utmost importance. Hence, for the first time a comparison between the amount of the total Se found in rice grains from 26 different genotypes cultivated for two consecutive agrarian years in the same open field and irrigated with the same water using three different water irrigation management techniques (i.e., the worldwide used continuous flooding irrigation, CF, and two intermittent techniques like saturation irrigation, SA and sprinkler irrigation, SP) has been performed. Data obtained substantiate the fact that the water management technique is effective in playing a major role in modulating the Se concentration in rice grain. In particular, the adoption of both the CF of SA irrigation techniques can be used when the aim is to increase the Se concentration in kernels, whereas the opposite result can be reached irrigating rice by SP, because this allows a reduction of the amount of this element of ca. 90% in comparison to the concentrations measured in rice irrigated by CF or SA techniques. Also the effect of the genotype can be used as a reliable additional tool in order to maximize the planned results, although the effect of the agrarian year might be able to modify, in an unpredictable albeit non-substantial way, the Se bioconcentration dynamics on the rice kernels. Although these results may envisage the way to reach an effective modulation of the Se amount in rice according the needs of specific people, additional experimentation should be done in order to achieve this very desirable goal.

## Figures and Tables

**Table 1:** Validation parameters for the ICP-MS determination of Se in rice.

LoD ^a^ (μg kg^−1^)	LoQ (μg kg^−1^)	Linearity	Repeatability ^b^		Intermediate Precision^c^		Trueness	
0.73	2.4	Concentration range (μg kg^−1^): 2.4–240	CRM rice flour	CV	CRM rice flour	CV	CRM (Concentration ± s ^e^, μg kg^−1^)	Recovery ^d^ (% ± s ^e^)
		Y = (a ± s_a_)X + (b ± s_b_) a = 0.0016; s_a_ = 0.0001; b = 0.00001; s_b_ = 0.00001; R^2^ = 0.9999	NIST 1568 ^a^	2.4	NIST 1568a	8.2	NIST 1568a (380 ± 40)	99 ± 2
			NCSZC 73008	3.5	NCSZC 73008	7.0	NCSZC 73,008 (61 ± 15)	100 ± 2
			NCSZC 11007	12	NCSZC 11007	14	NCSZC 11,007 (140 ± 20) ^f^	--
			IRMM 804	11	IRMM 804	20	IRMM 804 (60 ± 10) ^f^	--

^a^ The LoD value is measured according to [64]; ^b^ evaluated by analyzing four different CRM five times within the same analytical session; ^c^ evaluated by analyzing four different CRM ten times in five different analytical sessions within one month; ^d^ evaluated by analyzing three aliquots of CRM rice flour within the same analytical session; ^e^ standard deviation, ^f^ the data reported is not the certified concentration, but the Se amount measured by this method.

**Table 2:** Total Se amounts (μg kg^−1^ ± s) in grains of 26 rice genotypes cultivated along two consecutive crop years with three different irrigation methods: continuous flooding (CF), saturation (SA), and sprinkler (SP) irrigation.

	CF		SA		SP	
Rice Genotype	Year 1	Year 2	Year 1	Year 2	Year 1	Year 2
Aleramo	110 ± 60	96 ± 7	115 ± 10	110 ± 20	7 ± 1	6.6 ± 0.9
Antares	88 ± 3	80 ± 10	80 ± 20	110 ± 30	11 ± 2	13 ± 1
*Apollo*	100 ± 70	56 ± 4	110 ± 20	100 ± 10	11 ± 2	16.0 ± 0.7
Balilla	70 ± 30	50 ± 20	90 ± 15	90 ± 20	9 ± 3	9 ± 2
Brio	80 ± 25	72 ± 9	120 ± 20	110 ± 30	8 ± 2	9 ± 1
Carnaroli	80 ± 10	82 ± 4	130 ± 20	130 ± 25	11 ± 2	11 ± 1
Carnise	150 ± 60	100 ± 20	130 ± 10	120 ± 30	9 ± 3	8 ± 1
Cerere	80 ± 20	60 ± 5	110 ± 30	70 ± 30	9 ± 4	9 ± 2
CRV04	65 ± 30	56 ± 8	100 ± 30	63 ± 6	8 ± 3	6 ± 1.5
CRV108	85 ± 50	70 ± 20	90 ± 10	110 ± 30	5 ± 1	9.0 ± 0.7
CRV114	110 ± 40	80 ± 20	80 ± 20	130 ± 30	6 ± 4	11.5 ± 0.9
CRV 390	70 ± 35	70 ± 20	95 ± 25	130 ± 20	5 ± 2	7.9 ± 0.8
Galileo	110 ± 20	60 ± 10	90 ± 20	120 ± 40	7 ± 3	8 ± 2
Gloria	95 ± 40	80 ± 25	110 ± 20	150 ± 50	8 ± 3	13 ± 1
Luxor	95 ± 50	80 ± 10	90 ± 20	90 ± 10	6.6 ± 0.8	8 ± 1
Musa	100 ± 30	65 ± 20	110 ± 20	120 ± 30	8 ± 2	12 ± 1.5
*Oceano*	75 ± 20	60 ± 15	90 ± 30	90 ± 20	7 ± 1	9.6 ± 0.9
Opale	110 ± 40	50 ± 10	100 ± 30	110 ± 3	7 ± 3	11 ± 1
Orione	100 ± 50	70 ± 20	80 ± 20	100 ± 30	7.0 ± 0.7	7.5 ± 0.8
Ronaldo	90 ± 40	55 ± 20	70 ± 20	130 ± 30	9 ± 2	19 ± 2
*Salvo*	110 ± 50	60 ± 10	80 ± 30	120 ± 60	8 ± 2	10.3 ± 0.9
Selenio	110 ± 60	60 ± 20	80 ± 20	100 ± 30	8 ± 2	13.0 ± 0.9
*Sprint*	90 ± 15	90 ± 20	80 ± 10	130 ± 20	8 ± 4	12 ± 1.5
*Thaibonnet*	80 ± 20	70 ± 10	75 ± 20	100 ± 20	8.2 ± 0.5	6 ± 1
*Urano*	110 ± 40	90 ± 30	100 ± 20	130 ± 40	10 ± 4	13 ± 2
Virgo	100 ± 50	80 ± 9	70 ± 20	100 ± 10	6.5 ± 2	7 ± 1.5
Average Indica genotypes	94	71	89	112	9	11
Average Japonica genotypes	95	71	97	110	8	10
Average all genotypes	95	71	95	111	8	10

Each datum is the average of four different samples (one from each replication), and each sample is evaluated five times. s = standard deviation; Indica genotypes in *italic* character, all averages have been calculated on the raw data (i.e., data have not been subjected to any rounding aimed to harmonize their significant digits with those of the relevant SD).

**Table 3:** Chemical and hydrological parameters of the soils (layer depth: 0–20 cm, particle size: < 2 mm) involved in the experiment. CF, continuous flooding irrigation, SA, saturation irrigation, SP, sprinkler irrigation.

Parameter	Irrigation Methods		
	CF	SA	SP
pH	7.75	7.56	7.65
Eh ^a^ (mV vs. SCE)	−225	−100 ^b^; 390 ^c^	130 ^d^
Carbonates (% as CaCO_3_)	<0.01	<0.01	<0.01
Total nitrogen (%)	0.04	0.03	0.14
Organic carbon (%)	1.3	1.3	1.1
Assimilable phosphorous (mg kg^−1^ as P_2_O_5_)	111	112	64
Exchangeable potassium (mg kg^−1^ as K_2_O)	220	230	210
Field capacity (%, *v*/*v*)	^e^	^e^	34.6
Permanent wilting point (%, *v*/*v*)	^e^	^e^	20.4

All data reported were measured in the second year of experimentation. If otherwise not reported, all parameters were evaluated according to Gazzetta Ufficiale 248/99 S.O. 185 of the Italian Republic, 1999 [70]. ^a^ Eh was evaluated according to Pansu and Gautheyrou, 2006 [71]; ^b^ measured five hours after saturation; ^c^ measured just before saturation; ^d^ measured halfway through each sprinkler cycle; ^e^ parameter not measured.

## Supplementary Materials

The following are available online, Table S1: Fertilization and herbicide treatments performed on fields object of the experiment. CF, continuous flooding irrigation, SA, saturation irrigation, SP, sprinkler irrigation.
