# Supplementary material for: Tuning of the Amount of Se in Rice (Oryza sativa) Grain by Varying the Nature of the Irrigation Method: Development of an ICP-MS Analytical Protocol, Validation and Application to 26 Different Rice Genotypes
Source: Molecules. 2020 Apr 17;25(8):1861. doi: 10.3390/molecules25081861 (PMC7221937; doi:10.3390/molecules25081861)
Supplement: Supplementary file 1 [file molecules-25-01861-s001.pdf]

Article

Tuning of the amount of Se in rice grain (*Oryza Sativa*) at varying of the nature of the irrigation method: development of an ICP-MS analytical protocol, validation and application on 26 different rice genotypes.

Antonino Spanu [1], Ilaria Langasco [2], Massimiliano Valente [2], Mario Antonello Deroma [1], Nadia Spano [2], Francesco Barracu [1], Maria I. Pilo [2] and Gavino Sanna [2,*]

[1] Dipartimento di Agraria, Università degli Studi di Sassari, Via E. De Nicola, 1, 07100 Sassari, Italy; ant.spanu@gmail.com (A.S.); mderoma@uniss.it (M.A.D.), fbarracu@uniss.it (F.B.)
[2] Dipartimento di Chimica e Farmacia, Università degli Studi di Sassari, Via Vienna, 2, 07100 Sassari, Italy; ilangasco@uniss.it (I.L.), massimiliano.valente@hotmail.com (M.V.), nspano@uniss.it (N.S.); mpilo@uniss.it (M.I.P.), sanna@uniss.it (G.S.).
* Correspondence: sanna@uniss.it; Tel.: +39 079 229500

Supplementary material

Table 1S. Fertilization and herbicide treatments performed on fields object of the experiment. CF, continuous flooding irrigation, SA, saturation irrigation, SP, sprinkler irrigation.

**CF and SA**	**Treatments**
Pre-sowing fertilization	First year of the experiment: N_2, 135 kg ha^{-1}; P_2O_5, 92 kg ha^{-1}; K_2O, 50 kg ha^{-1}<br>Second year of th experiment: N_2, 63 kg ha^{-1}; P_2O_5, 92 kg ha^{-1}; K_2O, 50 kg ha^{-1}
Pre-emergent weed treatment	Both years of the experiment: *Pendimethalin*®, 1,320 g ha^{-1}
Post-emergent weed treatment	First year of the experiment: Penoxsulam, 43.8 g ha^{-1}, Triclopyr, 133.2 g ha^{-1} and *MCPA*®, 376 g ha^{-1}<br>Second year of the experiment: *Cinosulfuron*®, 80 g ha^{-1}
Coverage fertilization	First year of the experiment: three treatments, 36 kg ha^{-1} of N_2 each<br>Second year of the experiment: three treatments, 45 kg ha^{-1} of N_2 each

**SP**	**Treatments**
Pre-sowing fertilization	First year of the experiment: N_2, 105 kg ha^{-1}; P_2O_5, 92 kg ha^{-1}; K_2O, 50 kg ha^{-1}<br>Second year of the experiment: N_2, 63 kg ha^{-1}; P_2O_5, 92 kg ha^{-1}; K_2O, 50 kg ha^{-1}
Pre-emergent weed treatment	First year of the experiment: *Pendimethalin*®, 1,500 g ha^{-1}<br>Second year of the experiment: *Pendimethalin*®, 1,650 g ha^{-1}
Post-emergent weed treatment	First year of the experiment: *none*<br>Second year of the experiment: *MCPA*®, 784 g ha^{-1}
Coverage fertilization	Both years of the experiment: three treatments, 30 kg ha^{-1} of N_2 each

MCPA: 4-chloro-o-tolyloxyacetic acid; (4-chloro-2-methylphenoxy)acetic acid.
